# La carence en vitamine D chez l'adulte au Gabon: cas isolé ou problème méconnu?

**DOI:** 10.11604/pamj.2014.19.183.5372

**Published:** 2014-10-21

**Authors:** Marie-Pierrette Ntyonga-Pono

**Affiliations:** 1 Faculté de Médecine de Libreville, Service de Médecine interne A, Libreville, Gabon

**Keywords:** Vitamine D, carence, Gabon, équateur, Vitamin D, deficiency, Gabon, equator

## Abstract

La carence en vitamine D chez l'adulte est un sujet d'actualité à cause de ses multiples effets et de son extension de par le monde. Cependant elle est peu explorée au Gabon et en Afrique centrale en général. Le but de cet article qui rapporte trois cas documentés de carence en vitamine D chez l'adulte au Gabon, est d'attirer l'attention sur l'existence de ce problème même en zone équatoriale ensoleillée. Vu les implications de cette carence dans diverses pathologies osseuses, cardio-vasculaires, métaboliques, infectieuses, auto-immunes, néoplasiques..., des recherches plus approfondies sont nécessaires pour cerner le problème et prendre des mesures appropriées.

## Introduction

La carence en vitamine D est un sujet d'actualité avec plus de 20000 publications dans Pubmed d'une part à cause de sa globalisation, touchant plus d'un milliard d'individus de par le monde [1] et d'autre part de ses conséquences [2]. En effet cette carence favorise les pathologies osseuses d'où le nom initial d'ostéomalacie, [3] mais aussi les maladies cardio-vasculaires dont l'hypertension artérielle, les maladies métaboliques dont l'insulino résistance et le diabète de type 2, les maladies auto-immunes, infectieuses, certaines néoplasies et l'on continue de découvrir d'autres pathologies [1, 2]. Cependant les travaux de recherche en Afrique subsaharienne sont peu nombreux, les travaux portant sur quelques groupes et ne permettent pas d'avoir une idée réelle du problème [4]. Rappelons que la vitamine D, provient principalement de la peau, par la transformation d'un précurseur le 7 déhydro cholestérol sous l'effet des rayons ultra-violets B (UVB) fournis par le soleil [2]. Environ 30 minutes d'exposition au soleil chaque jour devraient suffire pour couvrir les besoins en vitamine D de l'adulte en Occident. Par contre chez les sujets à peau foncée, la mélanine agit comme un écran solaire diminuant l'absorption des UVB et réduisant la synthèse de vitamine D de 99% [1]. L'exposition solaire quotidienne devrait donc être plus prolongée, 3 à 5 fois plus chez les individus à peau foncée, pour combler les besoins en vitamine D [5]. Une plus faible partie est apportée par l'alimentation car peu d'aliments sont riches en Vitamine D. Ce sont essentiellement les poissons gras (thon, maquereaux, harengs, saumon), à un moindre degré le lait, le beurre les oeufs et certains végétaux comme les champignons [1, 2]. La mise en évidence de cette carence nécessite le dosage de la 25 Hydroxy vitamine D (25(OH) D) dont les valeurs normales se situent entre 30-40 ng/ml (75-100mmol/l). Entre 21- 29 ng/ml il y a insuffisance en vitamine D et en dessous de 20 ng/ml il y a carence [1]. Quant à l'intoxication, elle est plus rare et survient au delà de 150 ng/ml [1] (conversion ng/ml x 2,5 = valeur mmol/l) [4]. Chez l'enfant cette carence en vitamine D est responsable du rachitisme aux signes cliniques évocateurs [2, 4], mais chez l'adulte l'expression est polymorphe comme dans les trois cas que nous rapportons.

## Patient et observation

### Observation 1: cas index

Il s'agit d'une femme de 57 ans, professionnelle de la santé, travaillant dans un laboratoire, que nous suivions à Libreville pour une thyréopathie auto-immune (maladie de Basedow) apparue alors qu'elle avait 45 ans. Le traitement par les anti thyroidiens de synthèse était difficile à cause des complications hématologiques, stabilisées par le propyl thio uracile et les rechutes étaient fréquentes. Par ailleurs, elle présentait aussi une hypertension artérielle traitée par amlodipine. Cette patiente a commencé à se plaindre de douleurs musculaires diffuses, avec faiblesse musculaire que nous avons d'abord rattachées à son problème thyroïdien. Nous avons aussi évoqué une myasthénie mais le dosage des anticorps anti récepteurs de l'acétylcholine était négatif, ainsi qu’ une fibromyalgie. Le bilan phosphocalcique fait è Libreville était normal. C'est lors d'un séjour en France pour un traitement de sa thyroïde par l'iode radioactif que la carence en vitamine D a été objectivée. En France le bilan fait a montré une calcémie à 2,35 mmol/l (normale entre 2,15 et 2,57mmol/l), une phosphorémie à 1,23 mmol/l (normale entre 0,87 et 1,45 mmol/l). La 25(OH)D était à 9 ng/ml (normale entre 30 et 100ng/ml), le taux de parathormone (PTH) intacte était à 83 pg/ml (normale entre 15 et 55pg/ml). Un traitement par 100000U de Vitamine D toutes les deux semaines pendant 2 mois a été débuté en France. Actuellement elle a un traitement d'entretien de 100000U tous les 2 mois. L’évolution clinique a été favorable et tous les paramètres biologiques se sont normalisés.

### Second cas

Femme de 52 ans, cadre dans une administration qui a consulté pour douleurs musculaires diffuses, lombalgies chroniques, malaise général. Elle avait vu différents médecins et plusieurs bilans avaient été faits sans déceler une anomalie particulière. Les troubles avaient été rattachés à la péri ménopause. Dans ses antécédents, elle avait présenté une stérilité primaire liée à des myomes utérins. La myomectomie effectuée il ya plus de 15 ans lui a permis d'avoir 3 enfants. L'examen clinique systématique était normal et la pression artérielle était à 110/78 mmHg. Le bilan phospho-calcique a montré une calcémie à 2,3 mmol/l, une phosphorémie à 1, 3 mmol/l, une magnésémie à 0,80 mmol/l. Le dosage de la 25(OH) D était à 24,4 ng/ml. La PTH dosée en différé était à 64 pg/ml (normale entre 15 et 55pg/ml). Ces deux derniers dosages ont été faits en France. Par ailleurs, L'hémogramme était normal, il n'y avait pas de syndrome inflammatoire, le bilan thyroïdien était normal. Nous avons débuté un traitement par Vitamine D 6000U/j et calcium 1,5g/j. La surveillance par le dosage de la calcémie, la calciurie des 24h et la phosphorémie ne montre pas d'anomalies. Après 2 mois nous sommes passés au traitement d'entretien par 1000U de vitamine D par jour. L’évolution clinique est favorable.

### Troisième cas

Femme de 54 ans, cadre dans une administration, qui a consulté pour malaise général, vertiges par moments, céphalées, crampes surtout la nuit, lombalgies et sciatalgie gauche. Dans ses antécédents on note des problèmes osseux à type de sténose dégénérative de L3-S1 objectivée par une IRM du rachis lombaire, une césarienne il ya environ 10 ans à la suite de laquelle elle a été mise sous contraception progestative par une injection trimestrielle de Medroxyprogesterone acetate. A l'examen clinique l'anomalie retrouvée était une hypertension artérielle méconnue (170/100 mm Hg) aux deux bras. Le contrôle hors du cabinet médical dans une pharmacie a montré une pression artérielle à 140/90 mmHg. Les explorations biologiques ont montré un hémogramme normal, une dyslipidémie avec élévation importante des triglycérides 4,2 mmo/l (normale entre 0,40 et 1,82 mmol/l) et un Cholestérol total limite à 5,91 mmol/l (normale inférieure à 5,81mmol/l). La fonction rénale était normale, de même que le ionogramme sanguin, le bilan thyroïdien et le dosage des dérivés méthoxylés urinaires. La calcémie était à 2,24 mmol/l, la phosphorémie à 0,66 mmol/l et la 25(OH) D à 16,8 ng/ml (normale entre 30-80). La PTH dosée en différé était à 59 pg/ml (normale entre 15 et 55pg/ml). Le traitement a été commencé par 6000 U/j de Vitamine D associée à 1,5 g de calcium per os. Vu la labilité tensionnelle nous avons introduit un traitement par Perindopril (IEC) à faibles doses ajustables. La contraception cause possible de la dyslipidémie a été arrêtée en concertation avec son gynécologue, d'autant plus que la FSH et la LH étaient élevés lors du bilan (profil de ménopause) et la patient a été mise sous Oméga 3. L’évolution clinique a été favorable. Le résumé de ces trois observations est rapporté sur le Tableau 1.

**Tableau 1:** Résumé des observations

Patient	Age	Signes cliniques	Calcémie (mmol/l) 2,15 -2,57	Phosphoré-mie(mmol/l) 0,87 -1,45	25 OH D(ng/ml) 30-50	PTH (pg/ml) 15 -55
1	57	Maladie de Basedow, HTA Rhinite allergique Myalgies, crampes	2,35	1,23	9	83
2	52	Malaise général, myalgies, crampes lombalgies	2,3	1,3	24,4	64*
3	54	Céphalées Lombalgies, crampes, sciatalgie HTA labile,	2,24	0,66	16,8	59*

* Dosage différé; mmol/l = millimoles/litre; ng/ml = nannogrammes/ml; pg/ml= picogrammes/ml

## Discussion

Ces trois femmes présentaient toutes un déficit en vitamine D, sévère chez la première patiente et moindre dans les deux autres. La PTH était franchement élevée dans le premier cas, et un peu moins dans les deux autres cas mais elle a été dosée en différé après le début du traitement. Quel lien il y a-t-il entre ces trois femmes? Elles ont pour point commun d’être des cadres dans leur profession, passant la majeure partie de leur journée dans leur lieu de travail, véhiculées et donc insuffisamment exposées au soleil. Toutes avaient une calcémie plutôt basse mais dans les limites de la normale, résultats qui s'expliquent par l'hyperparathyroïdie secondaire, retrouvée dans les 3 cas, pour maintenir l'homéostasie du calcium [1].

Cette association baisse de la vitamine D et hyperparathyroïdie secondaire a déjà été décrite dans les populations noires aux USA, reliée à une certaine adaptation de ces populations déportées [6]. Mais des effets délétères liés à l’élévation de la PTH ont été décrits, notamment l'hypertension artérielle, l'ostéoporose [2] et l’élévation de la mortalité globale [7]. Deux de nos patientes présentent une hypertension artérielle, affection multifactorielle mais la carence en vitamine D peut y contribuer [2], notamment en favorisant l’élévation de la rénine plasmatique et la prolifération des cellules musculaires lisses. Une patiente avait des problèmes auto-immuns qui étaient peut être liés à cette carence sévère en vitamine D car le rôle de ce déficit dans la survenue de maladies auto-immunes été décrit par plusieurs auteurs [1, 2]. La troisième a souffert de fibromyomes utérins pathologie liée à des troubles hormonaux mais dans laquelle le déficit en vitamine D a été récemment incriminé [8]. Les signes cliniques évocateurs retrouvés chez nos patientes étaient la présence de myalgies, lombalgies et crampes, signes peu spécifiques mais retrouvés aussi par d'autres auteurs [9]. La calcémie, facilement demandée sous nos latitudes était plutôt basse, mais dans les limites de la normale dans les 3 cas, ce qui fait méconnaître le diagnostic car on associe carence en vitamine D à hypocalcémie du fait du rôle de la vitamine D dans l'absorption intestinale du calcium [1, 4]. Mais on ne pense pas à l'hyperparathyroïdie secondaire qui permet de puiser le calcium osseux, pour maintenir l'homéostasie calcique, au prix d'une certaine ostéoporose [1], non objectivée dans mon pays par manque d’équipement. C'est ce qui explique que le diagnostic ait été fait tardivement dans le cas index car nous n’étions pas sensibilisée à ce problème. Pour ce diagnostic, il faudrait demander le dosage de la 25 (OH) D couplé si possible à celui de la parathormone, mais ces dosages hormonaux ne sont malheureusement pas faits sur place, ce qui limite les explorations. Il faudrait aussi éliminer les autres causes d'hypocalcémie avec d'hyperparathyroïdie secondaire notamment rénales et digestives [1, 2]. Le traitement, simple, repose sur l'administration de Vitamine D soit par une dose de charge de 50000 U par semaine ou 100000 U toutes les deux semaines pendant 8 semaines [5], ou par une dose quotidienne entre 5000 et 10000 U par jour jusqu’à la correction de la carence [3]. Le traitement d'entretien doit ensuite être poursuivi soit par une dose quotidienne de 1000 à 2000 U par jour [3], soit par une dose de 100000 U tous les 2-3 mois [5]. Il est recommandé d'associer le calcium à la dose de 1 à 1,5 g par jour. Pour notre part nous avons préféré, en traitement d'attaque, donner une dose quotidienne de Vitamine D de 6000U/j entre 5000 et 10000 U/j[3]et un traitement d'entretien de 1000 U par jour en surveillant périodiquement les différents paramètres biologiques: calcémie, phosphorémie, calciurie des 24 heures et 25 OH D. En effet, le patient sensibilisé réalise davantage d'activités en plein air et nous ne souhaiterions pas arriver à une surcharge en vitamine D qui a aussi des effets délétères liés hypercalcémie au-delà de150 ng/ml [1, 2]. On peut bien sûr proposer de réaliser des activités en plein air, non pendant 30 minutes par jour mais pendant 3 fois plus de temps à cause de l'hyperpigmentation cutanée [5]. Cela semble difficilement réalisable pour certains travailleurs et des pays développés comme la France ont plutôt choisi la supplémentation en vitamine D entre 800 et 1500 Unités/jour chez l'adulte selon l’âge[2]

Ces patientes représentent-elles des cas isolés ou il y a-t-il un problème plus étendu mais méconnu? En effet les sociétés africaines connaissent des mutations rapides avec une urbanisation galopante incriminée dans la progression des maladies chroniques [4], notamment cardiovasculaires et métaboliques. Le Gabon fait partie de ces pays avec plus de 84% des habitants vivant en milieu urbain[4] et un grand nombre de fonctionnaires et bureaucrates, se déplaçant en taxis bon marché ou véhiculés, et donc peu exposés au soleil. Dans la transition épidémiologique que connaissent les pays en voie de développement, on évoque les changements dans l'alimentation, la sédentarité, l'obésité mais la carence en vitamine D n'est pas incriminée alors qu'elle pourrait intervenir dans le développement des maladies chroniques. En effet il a été montré que la carence en vitamine D favorise, entre autres, l'hypertension artérielle, l'insulino résistance tout comme l'obésité avec un rôle sur la survenue du diabète de type 2 [1, 2]. Par ailleurs des travaux récents de chercheurs de l'Inde où la prévalence de la carence en vitamine D touche environ 70% de la population ont montré un effet positif de la vitamine D contre la progression du diabète sucré [10], confirmant l'implication de cette carence dans cette pathologie.

Il est vrai que notre article ne porte sur quelque cas mais nos retrouvons un profil commun d'individus de classe moyenne ou aisée se déplaçant en voiture et travaillant dans des lieux fermés et par conséquent peu exposés au soleil même sous l’équateur. En effet, dans les pays tropicaux, différentes recherches ont montré des prévalences de cette carence en vitamine D de 30-50% voire 70% dans les pays du Moyen Orient et d'Asie beaucoup plus chez les femmes à cause de leur habillement et de leur manque d'exposition au soleil [1, 10]. Nous continuons à découvrir de plus en plus de cas confirmés en consultation externe, mais il serait souhaitable d'effectuer de véritables enquêtes épidémiologiques dans différentes catégories de la population en milieu urbain et rural pour cerner le problème, établir des normes nationales afin d'entreprendre des actions de prévention et d’éducation à grande échelle qui pourront avoir un impact sur l'explosion des maladies chroniques dans nos populations [10].

## Conclusion

Au travers de ces cas cliniques, on peut supposer que la carence en vitamine D existe probablement à une plus grande échelle dans certaines couches des populations urbaines d'Afrique Noire. Ses conséquences multiples favorisant diverses pathologies méritent qu'on se penche davantage sur ce problème. Il serait utile de faire des dosages de 25(OH) D à large échelle dans différentes couches de la population, pour cerner le problème, établir des normes locales et prendre des mesures pour corriger et prévenir cette carence.
